# On (Correctly Representing) Comprehension-Based Monitoring in Speaking: Rejoinder to Nozari (2020)

**DOI:** 10.5334/joc.112

**Published:** 2020-09-03

**Authors:** Ardi Roelofs

**Affiliations:** 1 Radboud University, Donders Institute for Brain, Cognition and Behaviour, Centre for Cognition, Nijmegen, NL

**Keywords:** conflict, self-monitoring, speaking, speech comprehension

## Abstract

Misunderstanding exists about what constitutes comprehension-based monitoring in speaking and what it empirically implies. Here, I make clear that the use of the speech comprehension system is the defining property of comprehension-based monitoring rather than conscious and deliberate processing, as maintained by Nozari (2020). Therefore, contrary to what Nozari claims, my arguments in Roelofs (2020) are suitable for addressing her criticisms raised against comprehension-based monitoring. Also, I indicate that Nozari does not correctly describe my view in a review of her paper. Finally, I further clarify what comprehension-based monitoring entails empirically, thereby dealing with Nozari’s new criticisms and inaccurate descriptions of empirical findings. I conclude that comprehension-based monitoring remains a viable account of self-monitoring in speaking.

## Introduction

According to the comprehension-based account of self-monitoring in speaking advanced by Levelt et al. (1999) and Roelofs (2004, 2005), speakers use their speech comprehension system for the monitoring of both external and internal speech. According to the production-based account of Nozari et al. (2011), speakers use their speech comprehension system for external monitoring, but internal monitoring is done by assessing the amount of conflict within the speech production system. In Roelofs (2020), I address the most important arguments of Nozari et al. and Nozari and Novick (2017) against a comprehension-based account of internal monitoring, and argue that none of the arguments are conclusive. In her response, Nozari (2020) maintains that the account that I defend is not one of comprehension-based monitoring because it lacks conscious and deliberate processing, and therefore is not suitable for addressing her criticisms. Moreover, she comes with new critique.

In this rejoinder, I clarify that the use of the speech comprehension system is the defining property of comprehension-based monitoring rather than conscious and deliberate processing. Therefore, my arguments in Roelofs (2020) are suitable for addressing the criticisms of Nozari et al. (2011) and Nozari and Novick (2017) against comprehension-based monitoring. Also, I make clear that Nozari (2020) does not correctly describe my view in a review of her paper. Finally, I further clarify what comprehension-based monitoring entails empirically, thereby addressing her new criticisms and inaccurate descriptions of empirical data.

## What is Comprehension-Based Monitoring?

Nozari (2020) states that “the model defended by Roelofs is not a comprehension-based monitor” (p. 2). According to her, this is because “Roelofs’s proposed model lacks a key feature of comprehension-based monitors, i.e., conscious and deliberate processing. … Thus this model is not suitable for addressing the criticisms raised against comprehension-based monitors” (p. 18).

However, unlike what Nozari maintains, “conscious and deliberate processing” is not a necessary feature of comprehension-based monitoring. Just like there are several possible production-based monitors and production-perception monitors (reviewed by Nozari, 2020), there are also several possible comprehension-based monitors. The defining property of a comprehension-based monitor is that it uses the speech comprehension system. Although conscious and deliberate processing is a feature of the comprehension-based monitor that Levelt (1983, 1989) proposes, it is not a necessary feature. The debate over production- vs. comprehension-based monitoring in the literature during the past few decades was not about whether self-monitoring is conscious and deliberate, but whether the speech comprehension system is used for internal monitoring (the comprehension-based account) or not (the production-based account). The model of Roelofs (2004, 2005) uses the speech comprehension system for internal monitoring, and therefore is an instance of a comprehension-based model, contrary to what Nozari maintains. Thus, the model is suitable for addressing the criticisms raised against comprehension-based monitors. Moreover, my arguments in Roelofs (2020) do not hinge on the particular model of Roelofs (2004, 2005) about how comprehension-based monitoring is accomplished (i.e., through comparisons between production and comprehension representations on all planning levels), as I make clear below.

Nozari (2020) also criticizes my proposal that comprehension-based monitoring is accomplished through condition-action rules, which implement procedural knowledge about how to achieve self-monitoring. She states, “if monitoring can be achieved without any such verification processes (and the alternative models of monitoring show that it can) proposing such mechanisms for monitoring is hard to justify” (p. 16).

However, alternatives like production-perception models require duplication of representations, which is problematic for the lemma level and higher. Moreover, alternatives like feedforward and feedback temporal models operate on activation levels only, making them very sensitive to distraction from self-produced or other-produced speech (see Roelofs, 1997, for extensive discussion). Spoken word planning through the use of condition-action rules solves the distraction problem (e.g., Roelofs, 1997, 2003), and it is only logical and parsimonious to assume that such rules are also used for self-monitoring. Condition-action rules are sometimes criticized for involving “sophisticated homunculi”, as Nozari (2020, p. 16) points out. Yet, a condition-action rule embodies a simple rather than “sophisticated” computation (e.g., Anderson et al., 2004; Meyer & Kieras, 1997). Moreover, work in theoretical neuroscience has demonstrated that the simple computations specified by condition-action rules may be realized by networks of spiking neurons (e.g., Eliasmith, 2013), making clear that the rules are not “homunculi”. A successful large-scale model of the functioning human brain (i.e., with 2.5 million neurons) has condition-action rules at its heart (Eliasmith et al., 2012). To conclude, the assumption of condition-action rules is not “hard to justify” theoretically and empirically.

## My View in a Review of Her Paper

Levelt (1989) states: “Talking as an intentional activity involves conceiving of an intention, selecting the relevant information to be expressed for the realization of this purpose, ordering this information for expression, keeping track of what was said before, and so on” (p. 9). He assumes that these processes are achieved by condition-action rules. In such a rule system, goals enable rule application (e.g., Anderson et al., 2004; Meyer & Kieras, 1997; Roelofs, 2003). Thus, if speakers want to monitor their speech for appropriateness or errors, a goal to do so has to be specified in working memory. But this does not imply, of course, that conceiving, selecting, ordering, keeping track, and monitoring are “the goal of speaking”. Instead, these mental processes take place in service of the overarching goal to communicate a message in an appropriate way and preferably without errors. Nevertheless, Nozari (2020) writes:

In his review of the current [i.e., her] paper, Roelofs clarified this by pointing out that in his view, monitoring is one of the goals of speaking (in addition to the communication goal) and suggested that condition-action rules are enabled by such a goal. If this is indeed the claim, then one must argue that monitoring performance is never the “goal” of any action. Speakers do not speak with the goal of detecting their errors; they speak with the goal of communicating a message (p. 16).

A problem here is that my view is not correctly described. Nozari (2020) states that speakers do not speak with the goal of detecting their errors, but speak with the goal of communicating a message. But, of course, they speak with the goal of communicating a message. The suggestion here is that I deny this. But why should I? What I said in my review is that self-monitoring is an intentional activity, that is, driven by a goal to do so (cf. Roelofs, 2004; Roelofs et al., 2007). Speakers have this goal in working memory. But this is different from saying that self-monitoring is the goal of speaking, which it is clearly not. Speakers may, for example, try to hide or misrepresent information and specify a goal in working memory to achieve this, but this does not mean that hiding or misrepresenting information is the goal of speaking.

To conclude, Nozari (2020) does not correctly describe what I said in my review of her paper. Comprehension-based monitoring does not entail that self-monitoring is the goal of speaking.

## The Cross-Talk Problem

According to Vigliocco and Hartsuiker (2002) and Huettig and Hartsuiker (2010), listening to internal speech while planning a word for production yields insurmountable cross-talk. Huettig and Hartsuiker state that “speakers can only ‘listen’ to internal speech when performing a silent task (like Özdemir et al.’s [2007] phoneme monitoring task), but not when speaking out loud” (p. 350). In Roelofs (2020), I argue that contrary to these claims, the empirical evidence suggests that speakers *can* listen to internal speech while talking aloud. In particular, Wheeldon and Levelt (1995) showed that participants can use internal speech to perform a monitoring task while at the same time producing overt speech. Moreover, cross-talk may cause a problem for the Dell-type model advanced by Nozari et al. (2011), as explained in Roelofs (1997), but not for the WEAVER++ model advanced by Levelt et al. (1999) and Roelofs (2004, 2005). In WEAVER++, selections are “threaded” (cf. Salvucci & Taatgen, 2008) through the use of condition-action rules rather than based on activation levels only. Rule-based selection solves the cross-talk problem.

In her response, still, Nozari (2020) suggests that my arguments are off the mark. She states that “the point relevant to Vigliocco and Hartsuiker’s (2002) criticism is whether concurrent production interferes with comprehension monitoring” (p. 19). However, the point is not the presence of interference (on which we agree) but whether speakers *can* do internal monitoring despite interference from producing overt speech. In Roelofs (2020), I point to empirical data showing that speakers can listen to internal speech while talking aloud, even in the presence of interference (i.e., Wheeldon & Levelt, 1995). Moreover, I point to the explanation by WEAVER++ of how speakers accomplish this feat (i.e., through condition-action rules).

Studies of speech comprehension have shown that hearing a spoken word (e.g., *beaker*) increases the number of gazes to phonologically related printed words (i.e., so-called cohort competitors like *beaver*) relative to unrelated words in a visual display. Huettig and Hartsuiker (2010) observed that this cohort effect occurred in picture naming after rather than before speech onset, which indicates that external rather than internal speech drove the gazes. This was taken as evidence against the monitoring of inner speech in an overt production task.

However, in Roelofs (2020), I point to evidence that speakers move gaze before speech onset only when the task requires this (e.g., Roelofs, 2007, 2008), but there was no such requirement in the study of Huettig and Hartsuiker (2010). This implies that their findings are neutral about comprehension-based monitoring. Nevertheless, in her response, Nozari (2020) states:

If this argument holds, then participants never have a reason to look at the cohort word, so a cohort advantage over unrelated items should never be observed in picture naming, but it was indeed present after naming the picture. … fixating the competitor is a non-deliberate action. (p. 20)

I agree that fixating the competitor is a non-deliberate action, but my point was that such non-deliberate action does not happen *before* speech onset. This implies that there was no reason to expect that gazes to cohort competitors would occur before speech onset in the study of Huettig and Hartsuiker. But this does not exclude that participants look at the cohort word after speech onset.

## Double Dissociation in Aphasia

Whereas Levelt et al. (1999) made the general claim that self-monitoring is achieved by listening to internal as well as external speech (following Levelt, 1983, 1989), in Roelofs (2004, 2005) I made the more specific proposal that comprehension-based monitoring is accomplished through comparisons between production and comprehension representations. However, my arguments in Roelofs (2020) do not depend on the particular proposal in Roelofs (2004, 2005) about how comprehension-based monitoring is accomplished. I illustrate this for the explanation of the double dissociation between comprehension and self-monitoring ability in aphasia.

Nozari et al. (2011) argue that the evidence for a double dissociation between comprehension and self-monitoring ability in persons with aphasia challenges comprehension-based monitoring. If speech comprehension is poor due to brain damage, then self-monitoring should also be poor because it is done using the impaired speech comprehension system. In Roelofs (2020), I argue that this reasoning only holds if comprehension and self-monitoring are identical processes, but this is not assumed by extant comprehension-based monitoring accounts. In Roelofs (2004, 2005), I propose that self-monitoring uses the speech comprehension system but also involves a comparison process. Similarly, Levelt (1989) assumes that “the monitor can compare the meaning of what was said or internally prepared to what was intended” (p. 13). Moreover, the comprehension system is directly internally fed by the production system in self-monitoring but not in comprehending others (Levelt et al., 1999; Roelofs, 2004, 2005; Roelofs et al., 2007). This may make self-monitoring and comprehending others differently sensitive to damage. As a consequence, under comprehension-based monitoring, a double dissociation between comprehension and self-monitoring ability may occur in patients with aphasia, contrary to what Nozari et al. maintain. Crucial for this account of the double dissociation is that internal links between production and comprehension are present within a speaker. The links are necessary for internally feeding production information to the comprehension system.

Surprisingly, in discussing the comprehension-based theory proposed by Levelt (1983, 1989), Nozari (2020) states:

It is important to note that this theoretical view … does not require structural connections between representations in production and perception within the speaker, as such connections are obviously absent between the perceptual system of a listener and the production system of another speaker (p. 4).

However, according to Levelt (1989) and Levelt et al. (1999), such structural connections are present within the speaker, because they are necessary for internally feeding production information to the comprehension system. Moreover, the mental lexicon is assumed to be shared between production and comprehension. This makes listening to internal speech different from listening to others. Because of the internal structural connections, and the direct sharing of information that they imply, self-monitoring and listening to others may be differentially sensitive to brain damage.

## Evidence Against Conflict Monitoring

As an alternative to comprehension-based monitoring of internal speech, Nozari et al. (2011) propose the assessing of conflict within the speech production system. Following a dominant account of action monitoring in nonlinguistic domains, they assume that conflict monitoring in speech production is done “most likely” (p. 9) by the anterior cingulate cortex (ACC). To the extent that the view of ACC conflict monitoring is empirically supported, the proposal of Nozari et al. gains credibility. However, in Roelofs (2020), I point to the mounting empirical evidence against the view of conflict monitoring by the ACC (these problematic findings are not mentioned by Nozari et al. and Nozari & Novick, 2017).

In her response, Nozari (2020) extensively criticizes one study with evidence against conflict monitoring (i.e., Burle et al., 2008) and she points for support of conflict monitoring to a study by Jiménez and Méndez (2013) investigating the congruency sequence effect (CSE). The latter is the observation that the difference in reaction time between incongruent and congruent trials is larger on post-congruent trials than on post-incongruent trials. Nozari states about the data of Jiménez and Méndez:

The direction of the CSE change, however, was in line with the predictions of the conflict-based account: a long series of low-conflict trials decreased the amount of control, leading to a larger CSE, whereas repeated encounters with high-conflict situations increased the amount of control, leading to a smaller CSE. (p. 25)

However, this is not what Jiménez and Méndez themselves claim to have observed. They state:

In other words, participants seem to be relying progressively more on the irrelevant features of the stimuli as the number of consecutively congruent trials increases, but they don’t seem to show an improvement toward avoiding the effect of such conflictive features with an increased number of consecutively incongruent trials. … Similar patterns of results have been recently obtained by Schlaghecken and Martini (2012) … and by Lamers and Roelofs (2011). (p. 282)

Lamers and Roelofs observed that the difference in reaction time between incongruent and congruent trials is larger on post-congruent trials than on post-incongruent trials and on post-neutral trials, which did not differ, challenging the conflict monitoring account. This pattern of effects was obtained both for the Eriksen flanker task and for the color-word Stroop task, and both for manual and vocal responding.

Replicating the reaction time patterns of Lamers and Roelofs (2011) in an EEG study, Compton et al. (2012) observed that during the inter-stimulus interval, alpha power was lower following congruent trials than following incongruent and neutral trials, which did not differ. This suggests that top-down control is actively adjusted following congruent trials (hence the lower alpha power), such that the attentional width on the next trial is increased. As a consequence, the distractor has a bigger impact during post-congruent than during post-incongruent and post-neutral trials, as reflected in the magnitude of the interference effect.

To summarize, there is accumulating evidence (briefly reviewed in Roelofs, 2020) that control adjustments are driven by expected or experienced congruent trials rather than by the response conflict evoked by incongruent trials, challenging the conflict monitoring account.

## Brain Activation During Monitoring

Gauvin et al. (2016) observed that when participants had to indicate by button press whether a self-produced or heard tongue twister contained an error, the ACC and other frontal areas were activated. Superior temporal gyrus (STG) was generally more active in the perception than in the production condition, and showed a complicated pattern of activations and de-activations in response to errors. Gauvin et al. took these findings to provide evidence against comprehension-based monitoring. However, in Roelofs (2020), I argue that the activation of the ACC and other frontal areas is expected under comprehension-based monitoring if executive control is involved and the ACC receives error signals (for WEAVER++ simulations, see Roelofs & Hagoort, 2002). Moreover, given that the perception system is activated differently in production than in listening to others, direct comparison between production and perception conditions in STG is expected to yield a complex pattern of results, especially if error-related activity is assessed. This corresponds to what Gauvin et al. observed.

However, in her response, Nozari (2020) disagrees with my description of the empirical neuroimaging findings of Gauvin et al. (2016). She states:

It appears to me that some important points are lost here. … In none of the comparisons did errors “activate” the superior temporal cortex; quite the contrary, when a reliable difference was found, it was in the direction of decreased activity of the STG during error production than correct production. (p. 31)

Yet, different from this claim by Nozari, Table 4 of Gauvin et al. lists, for error versus correct trials, a significant activation for perception (MNI xyz-coordinates: 52, –26, 4; *p* < .05) and both significant activations (xyz-coordinates: –58, 12, 4; *p* < .005) and deactivations (e.g., xyz-coordinates: –60, –18, 4; *p* < .005) for production. Without an explicit model of error monitoring in the production of tongue twisters under masking noise, the activations and deactivations are difficult to interpret. Deactivation may occur for various reasons. For example, errors may occur because words in a tongue twister are not enough activated during production, which when fed forward into the speech comprehension system may lead to under-activation of the comprehension system. As a consequence, the STG may be deactivated for error as compared to correct trials, as Gauvin et al. observed.

## On Parsimony in Explaining Self-Monitoring

At the end of her paper, Nozari (2020) argues that speaking is a complex process, and therefore requires various types of monitors, meeting different demands. However, in the beginning of her paper, she acknowledges that comprehension-based monitoring probably meets most of these demands. She states:

The account is also remarkable in its scope: since listeners try to extract meaning out of all aspects of an utterance (from speaker’s intentions to speech sounds), a comprehension-based monitor that operates in a similar manner, i.e., by trying to “listen” to the speaker’s internal speech, should also be capable of monitoring all aspects of communication. (p. 5)

Thus, given its completeness and parsimony, the view of comprehension-based monitoring should not be dismissed lightly, as Nozari does. In Roelofs (2020) and this rejoinder, I have indicated that, when comprehension-based monitoring and its empirical implications are correctly represented, no serious arguments exist against it.

## Conclusion

In this rejoinder, I made clear that the use of the comprehension system is the defining property of comprehension-based monitoring rather than conscious and deliberate processing, as Nozari (2020) maintains. Therefore, my arguments in Roelofs (2020) are suitable for addressing her criticisms raised against comprehension-based monitoring. Moreover, I further clarified what comprehension-based monitoring entails empirically, thereby dealing with the new criticisms raised by Nozari. I conclude that comprehension-based monitoring remains a viable account of self-monitoring in speaking.
